# Supplementary material for: Granulin loss and TMEM106B risk converge on lysosomal C-terminal fragment pathology in frontotemporal dementia
Source: bioRxiv. 2026 Mar 27:2026.03.25.713523. Preprint. [Version 2] doi: 10.64898/2026.03.25.713523 (PMC13042028; doi:10.64898/2026.03.25.713523)
Supplement: Supplement 1 [file NIHPP2026.03.25.713523v2-supplement-1.pdf]

bioRxiv preprint doi: https://doi.org/10.64898/2026.03.25.713523; this version posted March 27, 2026. The copyright holder for this preprint (which was not certified by peer review) is the author/funder, who has granted bioRxiv a license to display the preprint in perpetuity. It is made available under aCC-BY 4.0 International license.

Supplementary figures

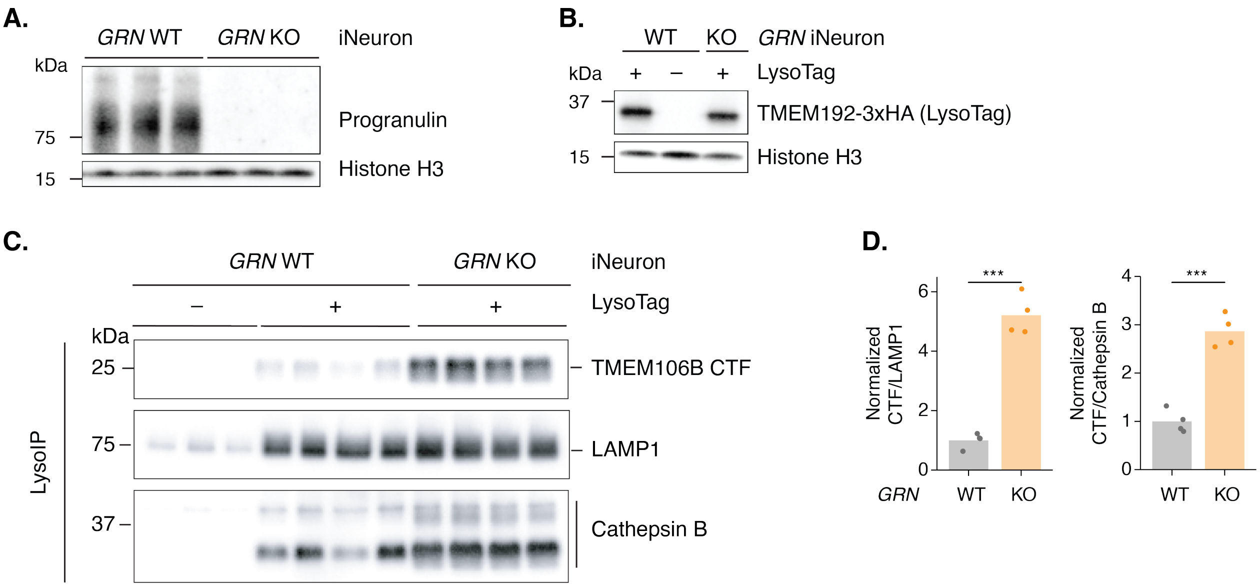

Figure S1. GRN loss increases levels of TMEM106B cleaved C-terminal fragments in the lysosome

A. Western blot confirms that *GRN* is knocked out in the *GRN* KO cell line.

B. Western blot confirms robust expression of the LysoTag (TMEM192-3xHA) in iNeurons.

C. Western blots show that *GRN* knockout (KO) in iNeurons leads to accumulation of TMEM106B C-terminal fragments (CTFs) in lysosomes. Purified lysosomes were from *GRN* wild-type (WT) iNeurons without LysoTag, *GRN* WT iNeurons with LysoTag, and *GRN* KO iNeurons with LysoTag. Samples were from the same experiment as in Figure 1A.

D. Quantification of TMEM106B CTFs from panel C. Normalized ratios were calculated by dividing the intensity of the TMEM106B CTF by either LAMP1 or Cathepsin B, then normalizing to the first bar (*GRN* WT with LysoTag for purified lysosomes).

Bar plots represent the mean, and each dot represents a replicate (n = 3 replicates per condition). Statistical significance was determined by a two-sided Welch's t-test: ns (not significant), $p > 0.05$; *, $p \leq 0.05$; **, $p \leq 0.01$; ***, $p \leq 0.001$; ****, $p \leq 0.0001$.

bioRxiv preprint doi: https://doi.org/10.64898/2026.03.25.713523; this version posted March 27, 2026. The copyright holder for this preprint (which was not certified by peer review) is the author/funder, who has granted bioRxiv a license to display the preprint in perpetuity. It is made available under aCC-BY 4.0 International license.

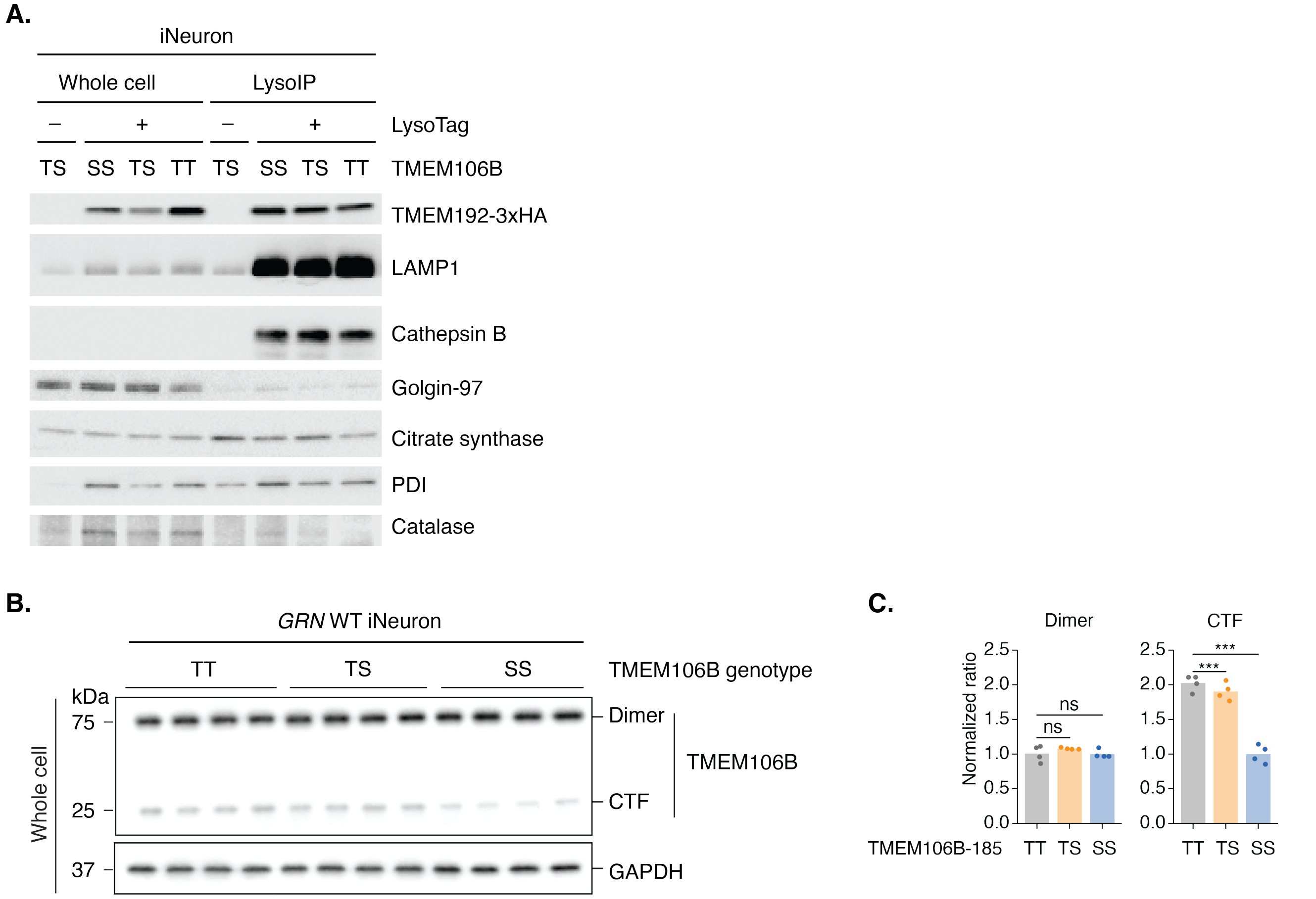

Figure S2. TMEM106B protective alleles are associated with reduced C-terminal fragment accumulation

A. Western blots show that LysoIP in iNeurons specifically purifies intact lysosomes without enriching other organelles. Western blots of whole cell lysates and purified lysosomes from LysoTag-expressing *GRN* WT iNeurons harboring different copy numbers of the TMEM106B protective coding variant (SS, TS, and TT) and *GRN* WT iNeurons harboring a heterozygous TMEM106B coding variant (TS) without a LysoTag as a negative control. Organelle markers are labeled.

B. Western blots show that the copy number of the protective S185 allele anti-correlates with TMEM106B CTF levels in iNeurons. Whole cell lysates were from *GRN* WT iNeurons with TT, TS, or SS genotypes.

C. Quantification of TMEM106B dimers and CTFs from panel B. Normalized ratios were calculated by dividing the intensity of each TMEM106B species (dimer or CTF) by the loading control (GAPDH), then normalizing to the first bar (TT genotype) of each TMEM106B species.

Bar plots represent the mean, and each dot represents a replicate (n =3 replicates per condition). Statistical significance was determined by two-sided Welch's t-test: ns (not significant), $p > 0.05$; *, $p \leq 0.05$; **, $p \leq 0.01$; ***, $p \leq 0.001$; ****, $p \leq 0.0001$.

bioRxiv preprint doi: https://doi.org/10.64898/2026.03.25.713523; this version posted March 27, 2026. The copyright holder for this preprint (which was not certified by peer review) is the author/funder, who has granted bioRxiv a license to display the preprint in perpetuity. It is made available under aCC-BY 4.0 International license.

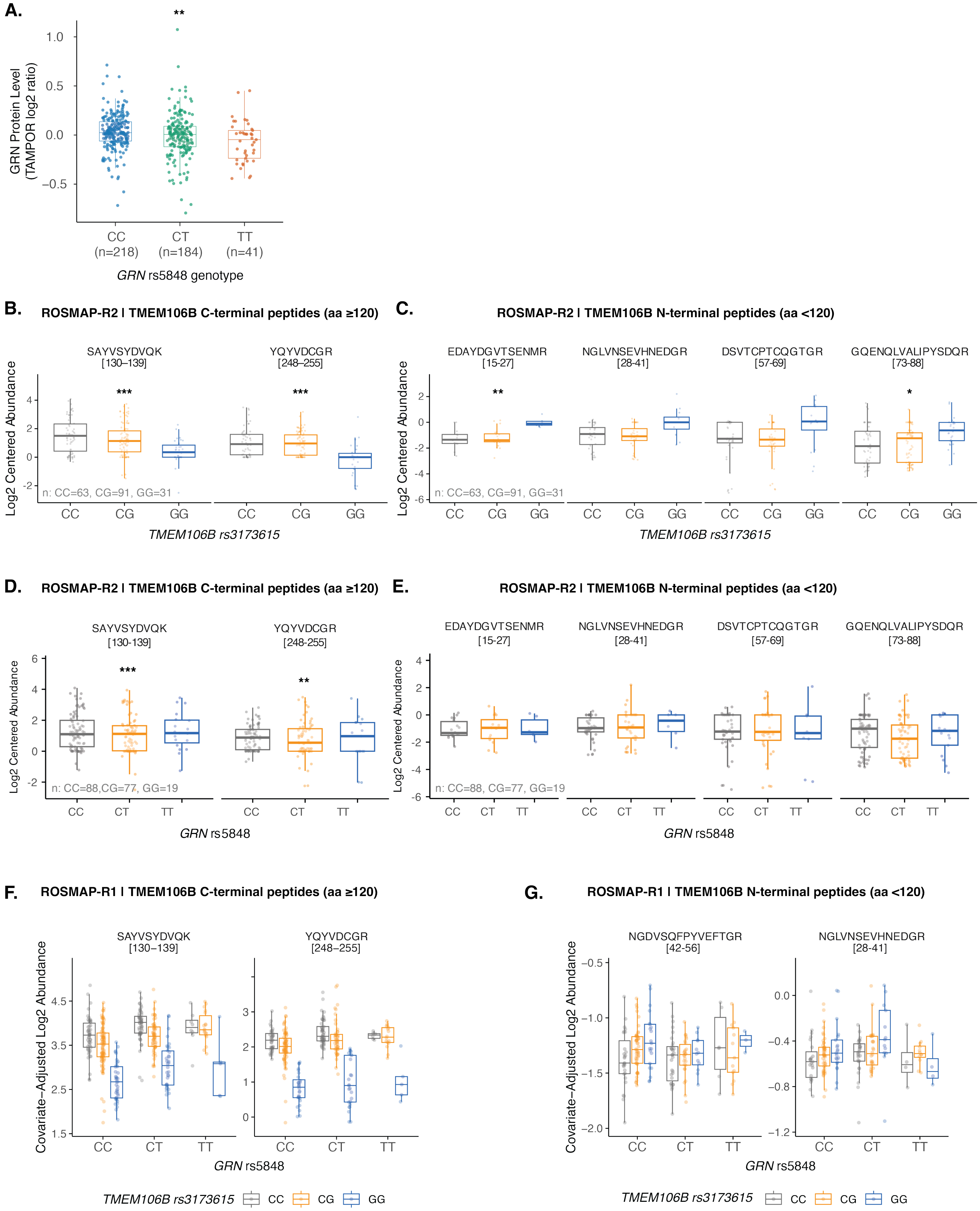

Figure S3. The TMEM106B risk haplotype and common GRN variant independently drive TMEM106B C-terminal fragment accumulation in human brain

bioRxiv preprint doi: https://doi.org/10.64898/2026.03.25.713523; this version posted March 27, 2026. The copyright holder for this preprint (which was not certified by peer review) is the author/funder, who has granted bioRxiv a license to display the preprint in perpetuity. It is made available under aCC-BY 4.0 International license.

A. Box plots showing that the common *GRN* risk variant (rs5848-T) is significantly associated with reduced progranulin protein levels. The association analysis was performed as using limma, adjusting for sex, postmortem interval, and final consensus cognitive diagnosis.
B. Box plots showing that the protective *TMEM106B* rs3173615 allele is significantly associated with reduced levels of peptides mapping to the TMEM106B C-terminal domain (CTD) in the second ROSMAP cohort (ROSMAP-R2). Association analysis was performed using a linear regression model adjusted for sample batch, sex, race, age of death, postmortem interval, and *APOE* genotype.
C. Box plots showing that the protective *TMEM106B* rs3173615 allele is significantly associated with increased levels of peptides mapping to the TMEM106B N-terminal domain (NTD) in the second ROSMAP cohort (ROSMAP-R2). The association analysis was performed as in panel B.
D. Box plots showing that the common *GRN* risk variant (rs5848-T) is not significantly associated with increased levels of TMEM106B CTD-mapping peptides in the second ROSMAP cohort (ROSMAP-R2). The association analysis was performed as in panel B.
E. Box plots showing that the common *GRN* variant (rs5848-T) is not significantly associated with levels of TMEM106B NTD-mapping peptides in the second ROSMAP cohort (ROSMAP-R2). The association analysis was performed as in panel B.
F. Covariate-adjusted box plots illustrating that the protective *TMEM106B* rs3173615 allele is associated with a stepwise reduction of CTD-mapping peptides across all *GRN* genotypes in in the first ROSMAP cohort (ROSMAP-R1), displaying the independent, additive effects of both variants.
G. Covariate-adjusted box plots showing N-terminal TMEM106B peptides across GRN genotypes in harmonized ROSMAP data.

Details of the statistical analysis can be found in the Methods section. Statistical significance was determined by two-sided Welch's t-test: ns (not significant), $p > 0.05$; *, $p \leq 0.05$; **, $p \leq 0.01$; ***, $p \leq 0.001$; ****, $p \leq 0.0001$.
